# A case of rapid-onset dystonia-parkinsonism accompanied by pyramidal tract impairment

**DOI:** 10.1186/s12883-016-0743-8

**Published:** 2016-11-11

**Authors:** Yanqiu Liu, Yan Lu, Xinqing Zhang, Shuping Xie, Tingting Wang, Tianwen Wu, Chaoyan Wang

**Affiliations:** 1 Department of Neurology, Xuanwu Hospital of Capital Medical University, Beijing, 100053 China; 2 Department of Neurology, The First Affiliated Hospital of Zhengzhou University, Zhengzhou, 450052 China; 3 Department of Magnetic Resonance, The First Affiliated Hospital of Zhengzhou University, Zhengzhou, 450052 China

**Keywords:** Rapid-onset dystonia-parkinsonism (RDP), Na^+^/K^+^ −ATPase α3 subunit gene (ATP1A3), E277K mutation, Pyramidal tract impairment

## Abstract

**Background:**

Rapid-onset dystonia-parkinsonism (RDP) is a rare autosomal dominant disorder that is caused by mutations in the ATP1A3 gene and is characterized by an acute onset of asymmetric dystonia and parkinsonism. To date, fewer than 75 RDP cases have been reported worldwide. Clinical signs of pyramidal tract involvement have been reported in several RDP cases, and none of them included the Babinski sign.

**Case presentation:**

We report a 24-year-old Chinese female with RDP who exhibited a strikingly asymmetric, predominantly dystonic movement disorder with a rostrocaudal gradient of involvement and parkinsonism. Physical examiniations revealed hyperactive reflexes, bilateral ankle clonus and positive Babinski sign in the right. DTI showed reduced white matter integrity of the corticospinal tract in the frontal lobe and subpontine plane. Genetic testing revealed a missense mutation of the ATP1A3-gene (E277K) in the patient.

**Conclusion:**

We suggest that pyramidal tract impairment could be involved in rapid-onset dystonia-parkinsonism and the pyramidal tract impairment in RDP needs to be differentiated from HSP.

**Electronic supplementary material:**

The online version of this article (doi:10.1186/s12883-016-0743-8) contains supplementary material, which is available to authorized users.

## Background

Rapid-onset dystonia-parkinsonism (RDP, DYT12) is a rare autosomal dominant movement disorder with variable expressivity and reduced penetrance that is caused by a mutation of ATP1A3 (Na^+^/K^+^ −ATPase α3 subunit gene) located on chromosome 19 [1]. To date, epidemiological data related to RDP is limited and fewer than 75 RDP cases have been reported worldwide [2].

RDP is characterized by the sudden onset of asymmetric dystonia and parkinsonism and presents a clear rostrocaudal gradient of symptoms (face > arms > legs) and prominent bulbar involvement [3, 4]. To our knowledge, clinical signs of pyramidal tract involvement have been reported in several RDP cases, but none of them included the Babinski sign. Although both the brisks reflexes and the ankle clonus exhibited in several RDP cases, scarce articles have discussed the pyramidal tract dysfunction in RDP. Here, we describe a Chinese RDP case with the Babinski sign, which indicates significant pyramidal tract impairment.

## Case presentation

**Fig. 1:**
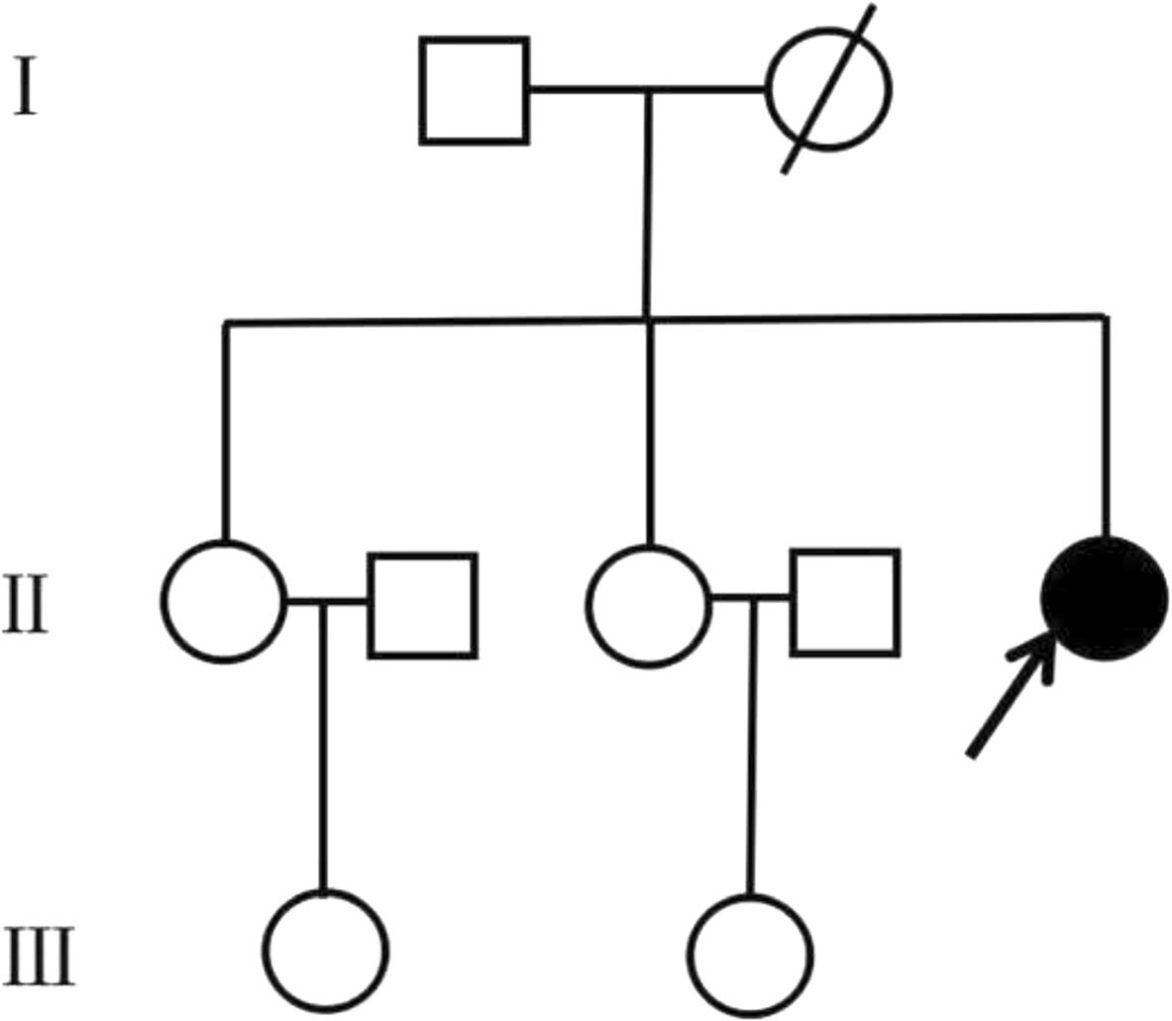
The pedgree for this patient. Squares indicate males; circles, females; arrow, proband; filled circle, affected female; circle with oblique line, died female

A 24-year-old Chinese female with severe speech and swallowing difficulties, prominent lower lip concavity, and walking difficulties was admitted to our institution in July 2015. In 2006, she displayed abnormal right leg posture while running. She was unaware of the abnormality until her father told her. In 2012, she suffered from right leg spasms while sleeping as well as notable gait disturbance. In April 2015, she displayed dragging of the right leg with involuntary inversion of the right ankle and slowness of movement. In May 2015, she experienced an acute onset of severe speech and swallowing difficulties, and these symptoms were followed by drooling, prominent lower lip concavity and dystonic right hand postures several days later. The symptoms slightly progressed over 1 week and then stabilized. Subsequently, her symptoms fluctuated frequently over a 1 week period. Depression and anxiety could worsen her symptoms. The patient presented with global developmental delay since early childhood and exhibited no preceding illness or drug use. There was no family history of dystonia or Parkinson’s disease (Fig. 1). The patient’s mother died of cerebrovascular disease at 30 years old.

**Fig. 2:**
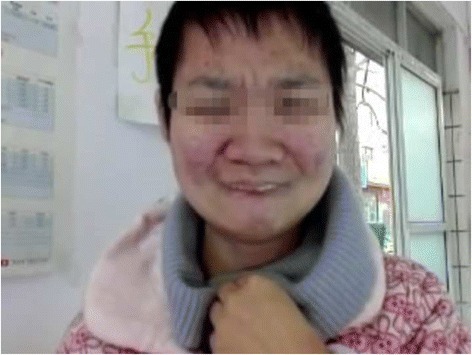
Prominent lower lip concavity in our patient. Our patient presented prominent lower lip concavity, as shown in the picture

The patient displayed prominent lower lip inversion (Fig. 2). Due to severe hypokinesia of the tongue she was anarthric. The muscle strength was normal. She presented cogwheel rigidity of her right arm and both legs, particularly the right leg. She also showed markedly slow movements, including finger and feet tapping. Deep tendon reflexes of the legs were hyperactive. Ankle clonus was present bilaterally with more than 3 cloni. The right foot displayed a positive Babinski sign, whereas the left displayed a suspicious positive.

**Fig. 3:**
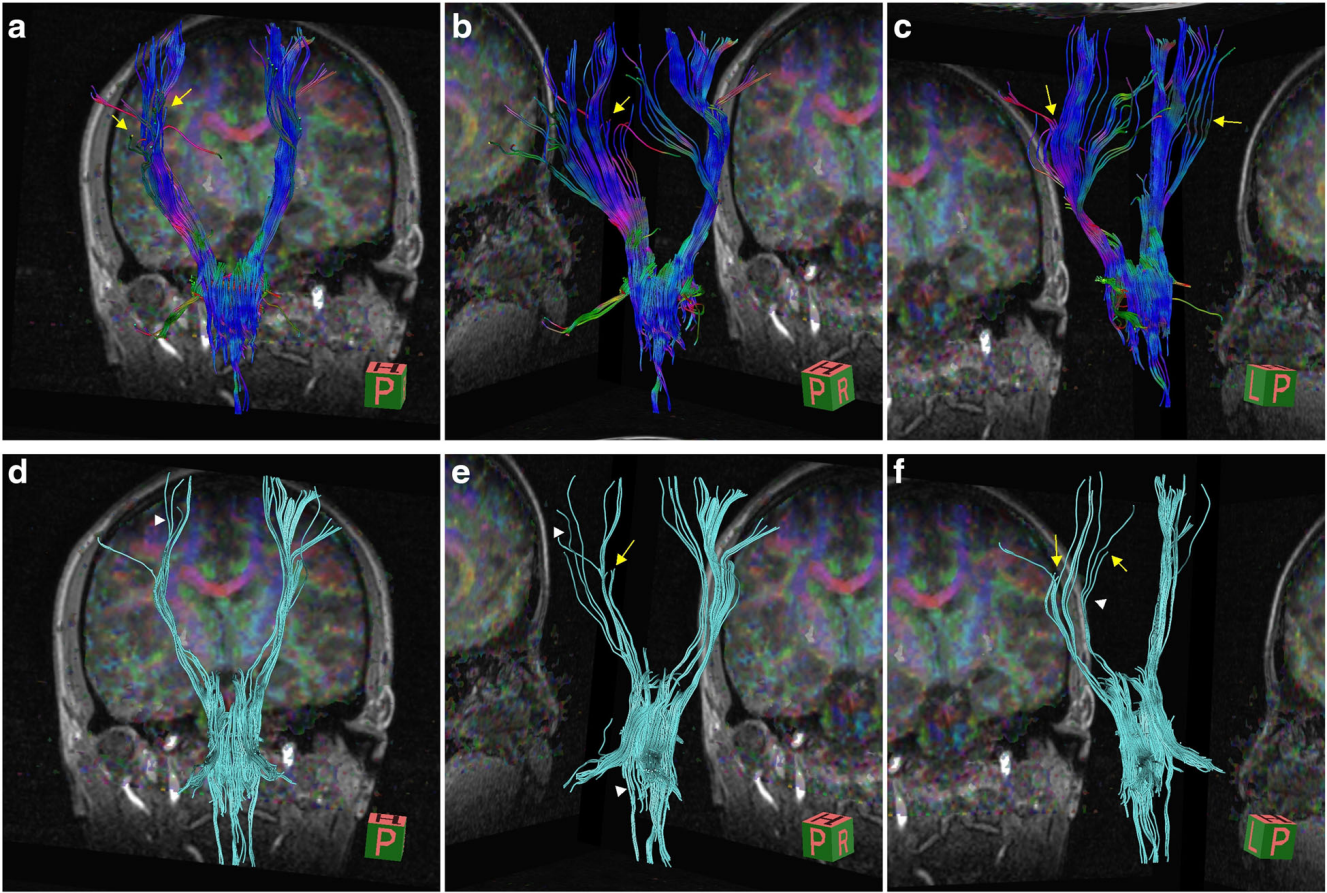
Diffusion tensor imaging (DTI) of the patient. Diffusion tensor imaging (DTI) showed reduced *white* matter integrity of the corticospinal tract in the frontal lobe and subpontine plane. The left side of corticospinal tract showed more sparse in the frontal lobe and subpontine plane than the other side (filled triangles in *white* shown in picture **d**, **e**, **f**). Also, we could find some interruption in left side of corticospinal tract (arrows in *yellow* in picture **a**, **b**, **c**, **e**, **f**). In addition, we could still see some interruption in the right side of corticospinal tract (arrows in *yellow* in picture **c**)

**Fig. 4:**
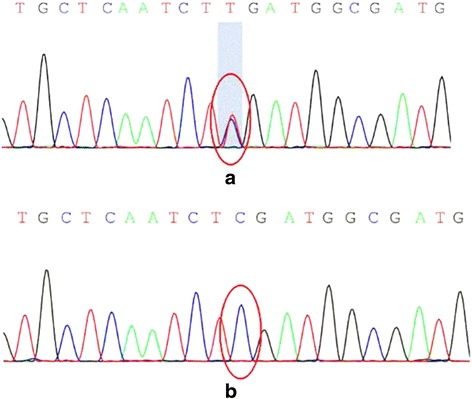
Next-generation sequencing of the patient and her father. Next-generation sequencing of the patient revealed a heterozygous nucleotide substitution (c.829G > A) in ATP1A3 gene in exon 8 of chromosome 19 (chr19:42489234) (**a**). Next-generation sequencing of the patient’s father revealed no nucleotide substitution in ATP1A3 gene (**b**)

Both laboratory tests and routine cerebrospinal fluid (CSF) studies were normal. Diffusion tensor imaging (DTI; Fig. 3) showed reduced white matter integrity of the corticospinal tract in the frontal lobe and subpontine plane. Genetic tests for spinocerebellar ataxia (SCA) and hereditary spastic paraplegia (HSP) yielded negative results (HSP-associated and SCA associated genes included in the gene sequencing of the patient were shown in Additional file 1.). Next-generation sequencing of the patient revealed a heterozygous nucleotide substitution (c.829G > A) in ATP1A3 gene in exon 8 of chromosome 19, resulting in an amino acid substitution of glutamic acid (E) to lysine (K) at codon position 277 (p.E277K), which her father did not carry (Fig. 4) (Dystonia-associated genes included in the gene sequencing of the patient were shown in Additional file 2).

The symptoms appeared to be slightly improved by the administration of trihexyphenidyl and baclofen. When she was admitted at our institution again 4 months later, ankle clonus and a positive Babinski sign were not observed, but deep tendon reflexes of the legs remained brisk.

## Conclusion

RDP (OMIM #128235), which was first reported by Dobyns in 1993 [5], has been linked to various mutations in the ATP1A3 [1, 3], which encodes the α3 subunit of the Na^+^/K^+^ −ATPase. The Na^+^/K^+^ −ATPase (sodium pump), which pumps three molecules of Na + out of and two molecules of K+ into cells, is highly expressed in the basal ganglia, cerebellum, thalamic nuclei, hippocampus and several areas of the pons [6] and plays an important role in maintaining the electrochemical gradient at the plasma membrane prior to depolarization [7]. ATP1A3 gene has been involved in five clinical neurological entities [8]: (1) RDP; (2) alternating hemiplegia of childhood (AHC); (3) CAPOS (cerebellar ataxia, areflexia, pes cavus, optic atrophy, sensorineural hearing loss) syndrome; (4) early infantile epileptic encephalopathy; and (5) Relapsing encephalopathy with cerebellar ataxia (RECA).

To date, 12 missense mutations, two 3-bp deletions and one 3-bp insertion in the ATP1A3 gene have been reported to be associated with RDP [4, 9]. The patient we presented had the E277K mutation, which has been previously reported in three individuals with adult-onset RDP [1, 3, 10]. RDP-associated ATP1A3 mutations are inherited in an autosomal dominant fashion but also frequently occur de novo [3, 4, 11]. We could not determine whether the patient’s mutation appeared de novo because the patient’s mother was deceased. However, there was no family history of dystonia or Parkinson disease, suggesting that our patient is likely a sporadic case.

Our patient displayed prominent lower lip inversion, a symptom that has not been described in previously reported RDP cases and was probably caused by the orofacial dystonia [3, 4, 10]. The patient had severe bulbar symptoms (dysarthria and dysphagia) and mild dystonia in her right arm and leg, consistent with the rostrocaudal gradient and asymmetric involvement of symptoms (face > arm > leg) in RDP [1, 3, 4].

About 13 reported RDP cases showed brisk reflexes on physical exammination, with only 2 cases performed ankle clonus simultaneously, but none of them exhibited the Babinski sign [5, 10, 12–16]. Although both the brisks reflexes and the ankle clonus exhibited in several RDP cases, scarce articles have discussed the pyramidal tract dysfunction in RDP [2]. However, Oblak et al. [2] reported neuropathological investigations in 4 siblings with RDP and described the involvement of corticospinal tract in RDP. Also, recent DTI studies have suggested that altered microstructural integrity of brainstem pathways and adverse interactions between the cerebellum and basal ganglia may contribute to the development of idiopathic early-onset dystonia [17, 18]. Our patient exhibited both ankle clonus and a positive Babinski sign, which were caused by impairment of the pyramidal tract. DTI in our patient revealed decreased white matter integrity of the corticospinal tract in the frontal lobe and subpontine plane, providing evidence of pyramidal tract impairment.

The pyramidal tract impairment in the patient needs to be differentiated from hereditary spastic paraplegia (HSP). In the diagnosis of our patient, we realized the pyramidal tract impairment and the dystonia in our patient at first, which indicates the probable diagnosis of HSP. As complicated HSP could encompass the parapyramidal signs (including dystonia), for example in SPG21,SPG35,SPG56 [19, 20], so we take the next-gene sequencing on HSP. And the gene test of HSP was negative, which excluded SPG1-39,SPG41-56,SPG61-74,SCA5,SCA7-16,SCA19,SCA22-23, SCA26-28, SCA34-36 and so on (see in Additional file 1).

However, approximately 4 months after the diagnosis was made and a treatment with baclofen and trihexyphenidyl was started, and when our patient was re-admitted to our clinic, she not only got better in drooling and walking, but also could speak single syllable. But the psychiary symptoms, such as anxiety, depression, were much worse. She even had violent behaviours sometimes. On neurological examination, the ankle clonus and Babinski sign were no longer present, but the deep tendon reflexes of the legs were hyperactive. Baclofen is a GABA-B autoreceptor agonist that has been used to treat spasticity [21] and dystonia [22], and trihexyphenidyl is an anticholinergic agent that has proven effective for the symptomatic treatment of dystonia in young patients [23]. Furthermore, Almeida GL et al. reported an 11-year-old boy with spastic diplegia cerebral palsy and found that ankle clonus and Babinski reflexes could be decreased during balofen administration [24]. The treatment with baclofen and trihexyphenidyl could have contributed to the resolution of the ankle clonus and Babinski sign; however, the real reason is unknown.

No curative treatment for RDP is currently available [1, 3]. The trihexyphenidyl and baclofen treatment provided limited benefits to the patient. In the future, therapies aimed at the Na^+^/K^+^-ATPase pump may be effective treatments for RDP.

## Additional files

Additional file 1: HSP-associated and SCA-associated genes included in the gene sequencing of the patient. (DOC 98 kb)
Additional file 2: Dystonia-associated genes included in the gene sequencing of the patient. (DOC 40 kb)

## Abbreviations

ATP1A3: Na^+^/K^+^ −ATPase α3 subunit gene
DTI: Diffusion tensor imaging
HSP: Hereditary spastic paraplegia
RDP: Rapid-onset dystonia-parkinsonism
