# Supplementary material for: A case of rapid-onset dystonia-parkinsonism accompanied by pyramidal tract impairment
Source: BMC Neurol. 2016 Nov 11;16:218. doi: 10.1186/s12883-016-0743-8 (PMC5105251; doi:10.1186/s12883-016-0743-8)
Supplement: Additional file 1: — HSP-associated and SCA-associated genes included in the gene sequencing of the patient. (DOC 98 kb) [file 12883_2016_743_MOESM1_ESM.doc]

**HSP-associated genes included in the gene sequencing of the patient.**

| Subtype | Gene | Mode of Inheritance | Subtype | Gene | Mode of Inheritance |
| --- | --- | --- | --- | --- | --- |
| SPG1 | L1CAM | XR | SPG35 | FA2H | AR |
| SPG2 | PLP1 | XR | SPG36 | unkonwn | AD |
| SPG3 | ATL1 | AD | SPG37 | unkonwn | AD |
| SPG4 | SPAST | AD | SPG38 | unkonwn | AD |
| SPG5 | CYP7B1 | AR | SPG39 | PNPLA6 | AR |
| SPG6 | NIPA1 | AD | SPG41 | unkonwn | AD |
| SPG7 | SPG7 | AR | SPG42 | SLC33A1 | AD |
| SPG7 | REEP2 | AD/AR | SPG43 | C19orf12 | AR |
| SPG8 | KIAA0196 | AD | SPG44 | GJC2 | AR |
| SPG9 | unkonwn | AD | SPG45 | unkonwn | AR |
| SPG10 | KIF5A | AD | SPG46 | GBA2 | AR |
| SPG11 | SPG11 | AR | SPG47 | AP4B1 | AR |
| SPG12 | RTN2 | AD | SPG48 | AP5Z1 | AR |
| SPG13 | HSPD1 | AD | SPG49 | TECPR2 | AR |
| SPG14 | unkonwn | AR | SPG50 | AP4M1 | AR |
| SPG15 | ZFYVE26 | AR | SPG51 | AP4E1 | AR |
| SPG16 | unkonwn | XR | SPG52 | AP4S1 | AR |
| SPG17 | BSCL2 | AD | SPG53 | VPS37A | AR |
| SPG18 | ERLIN2 | AR | SPG54 | DDHD2 | AR |
| SPG19 | unkonwn | AD | SPG55 | C12orf65 | AR |
| SPG20 | SPG20 | AR | SPG56 | CYP2U1 | AR |
| SPG21 | [SPG21](http://www.genenames.org/cgi-bin/gene_symbol_report?match=SPG21) | AR | SPG61 | ARL6IP1 | AR |
| SPG22 | SLC16A2 | XR/XD | SPG62 | ERLIN1 | AR |
| SPG24 | unkonwn | AR | SPG63 | KIF1C | AR |
| SPG25 | unkonwn | AR | SPG64 | USP8 | AR |
| SPG26 | unkonwn | AR | SPG65 | WDR48 | AR |
| SPG26 | B4GALNT1 | AR | SPG66 | AMPD2 | AR |
| SPG27 | unkonwn | AR | SPG67 | ENTPD1 | AR |
| SPG28 | DDHD1 | AR | SPG68 | NT5C2 | AR |
| SPG29 | unkonwn | AD | SPG69 | ARSI | AR |
| SPG30 | KIF1A | AR | SPG70 | PGAP1 | AR |
| SPG31 | REEP1 | AD | SPG71 | FLRT1 | AR |
| SPG32 | unkonwn | AR | SPG72 | RAB3GAP2 | AR |
| SPG33 | ZFYVE27 | AD | SPG73 | MARS | AR |
| SPG34 | unkonwn | XR | SPG74 | ZFR | AR |
| SPG | MAG | AR | SPG+HSN | FAM134B | AR |
| SPG | BICD2 | AR | SPG+HSN | CCT5 | AD |
| SPG | LYST | AR/AD |  |  |  |

**SCA-associated genes included in the gene sequencing of the patient.**

| Subtype | Gene | Mode of Inheritance |
| --- | --- | --- |
| SCAR1(AOA2) | Senataxin | AR |
| SCAR7(Childhood onset) | TPP1 | AR |
| SCAR8(ARCA1) | SYNE1 | AR |
| SCAR9(ARCA2) | CABC1 | AR |
| SCAR10(Ataxia + Motor PN) | ANO10 | AR |
| SCAR11(ARCA + MR) | SYT14 | AR |
| SCAR12(Epilepsy + MR) | WWOX | AR |
| SCAR13 | GRM1 | AR |
| SCAR14(Infant onset) | SPTBN2 | AR |
| SCAR15 | KIAA0226 | AR |
| SCAR16 | STUB1 | AR |
| SCA5 | SPTBN2 | AD |
| SCA11 | TTBK2 | AD |
| SCA 13 | KCNC3 | AD |
| SCA14 | PRKCG | AD |
| SCA15 | ITPR1 | AD |
| SCA16 | ITPR1 | AD |
| SCA19 | KCND3 | AD |
| SCA22 | KCND3 | AD |
| SCA23 | PDYN | AD |
| SCA26 | EEF2 | AD |
| SCA27 | FGF14 | AD |
| SCA28 | AFG3L2 | AD |
| SCA34 | ELOVL4 | AD |
| SCA35 | TGM6 | AD |
| SCA36 | NOP56 | AD |
