# Supplementary material for: A case of rapid-onset dystonia-parkinsonism accompanied by pyramidal tract impairment
Source: BMC Neurol. 2016 Nov 11;16:218. doi: 10.1186/s12883-016-0743-8 (PMC5105251; doi:10.1186/s12883-016-0743-8)
Supplement: Additional file 2: — Dystonia-associated genes included in the gene sequencing of the patient. (DOC 40 kb) [file 12883_2016_743_MOESM2_ESM.doc]

**Dystonia-associated genes included in the gene sequencing of the patient**

| Subtype | Gene | Mode of Inheritance |
| --- | --- | --- |
| DYT1 | TOR1A | AD |
| DYT2 | Unknown | AR |
| DYT4 | TUBB4A | AD |
| DYT6 | THAP1 | AD |
| DYT7 | Unknown | AD |
| DYT13 | Unknown | AD |
| DYT17 | Unknown | AR |
| DYT21 | Unknown | AR |
| DYT23 | CIZ1 | AD |
| DYT24 | ANO3 | AD |
| DYT25 | GNAL | AD |
| DYT9 | SCL2A1 | AD |
| DYT10 | PRRT2 | AD |
| DYT8 | MR1 | AD |
| DYT19 | Unknown | AD |
| DYT20 | Unknown | AD |
| DYT11 | SGCE | AD |
| DYT12 | ATP1A3 | AD |
| DYT16 | PRKRA | AR |
| DYT5 | GCH1 | AD/AR |
| THD | TH | AR/AD |
| DYT15 | Unknown | AD |
| DYT, myoclonic | DRD2 | AR/AD |
| DYT3 | TAF1 | XR |
